# Assessing the Functional Role of Frontal Eye Fields in Voluntary and Reflexive Saccades Using Continuous Theta Burst Stimulation

**DOI:** 10.3389/fnins.2018.00944

**Published:** 2018-12-14

**Authors:** Seref Can Gurel, Miguel Castelo-Branco, Alexander T. Sack, Felix Duecker

**Affiliations:** ^1^Brain Stimulation and Cognition Group, Department of Cognitive Neuroscience, Faculty of Psychology and Neuroscience, Maastricht University, Maastricht, Netherlands; ^2^Maastricht Brain Imaging Center, Maastricht, Netherlands; ^3^Department of Psychiatry, Faculty of Medicine, Hacettepe University, Ankara, Turkey; ^4^Coimbra Institute for Biomedical Imaging and Translational Research, Coimbra, Portugal

**Keywords:** frontal eye field, continuous theta burst stimulation, reflexive saccades, voluntary saccades, oculomotor control

## Abstract

The frontal eye fields (FEFs) are core nodes of the oculomotor system contributing to saccade planning, control, and execution. Here, we aimed to reveal hemispheric asymmetries between left and right FEF in both voluntary and reflexive saccades toward horizontal and vertical targets. To this end, we applied fMRI-guided continuous theta burst stimulation (cTBS) over either left or right FEF and assessed the consequences of this disruption on saccade latencies. Using a fully counterbalanced within-subject design, we measured saccade latencies before and after the application of cTBS in eighteen healthy volunteers. In general, saccade latencies on both tasks were susceptible to our experimental manipulations, that is, voluntary saccades were slower than reflexive saccades, and downward saccades were slower than upward saccades. Contrary to our expectations, we failed to reveal any TMS-related effects on saccade latencies, and Bayesian analyses provided strong support in favor of a TMS null result for both tasks. Keeping in mind the interpretative challenges of null results, we discuss possible explanations for this absence of behavioral TMS effects, focusing on methodological differences compared to previous studies (task parameters and online vs. offline TMS interventions). We also speculate about what our results might reveal about the functional role of FEF.

## Introduction

The frontal eye field (FEF) is a core node of the oculomotor system, interacting with cortical and subcortical structures during saccade planning, control, and execution. Neuroimaging studies have consistently reported bilateral BOLD signal increases near the junction of the precentral and superior frontal sulcus when tasks require saccadic eye movements (Darby et al., 1996; Corbetta et al., 1998; Berman et al., 1999; Beauchamp et al., 2001; Rosano et al., 2002; Grosbras et al., 2005) and patients with lesions to these brain regions are often characterized by impaired oculomotor function such as gaze deviations (Singer et al., 2006) and altered saccade parameters (Guitton et al., 1985; Pierrot-Deseilligny et al., 1987, 1991; Henik et al., 1994; Rivaud et al., 1994; Gaymard et al., 1999; Machado and Rafal, 2004a,b). Similarly, transcranial magnetic stimulation (TMS) applied over FEF in healthy volunteers has been shown to affect saccade latencies (Vernet et al., 2014).

On a general level, these research lines represent converging evidence in support of a causal role of FEF in saccadic behavior, however, a more detailed understanding of its exact role with regard to different aspects of saccade generation is still missing. This is partly due to discrepancies between studies that seem difficult to reconcile (for reviews, see Muri and Nyffeler, 2008; Vernet et al., 2014). To illustrate, various studies investigated latencies of reflexive saccades in patients with FEF lesions. When using gap paradigms (tasks in which the fixation point is turned off before target presentation), some experiments found normal saccade latencies (Pierrot-Deseilligny et al., 1991; Rivaud et al., 1994; Gaymard et al., 1999), whereas others reported either increased (Pierrot-Deseilligny et al., 1987) or decreased saccade latencies (Henik et al., 1994). When using an overlap paradigm (tasks in which the fixation point is still on before target presentation), increased saccade latencies have been consistently reported, yet, this impairment was either observed for saccades toward both hemifields (Rivaud et al., 1994) or explicitly limited to the contralesional (Gaymard et al., 1999) or ipsilesional hemifield (Machado and Rafal, 2004b). Importantly, such discrepancies are by no means limited to studies investigating reflexive saccades; equally heterogeneous results have been found for voluntary saccades (Henik et al., 1994; Rivaud et al., 1994; Gaymard et al., 1999) and anti-saccades as well (Guitton et al., 1985; Rivaud et al., 1994; Gaymard et al., 1999; Machado and Rafal, 2004a). The extent and localization of brain lesions most likely contribute to the observed differences in findings. Additionally, the lack of a clear understanding about functional reorganization and compensatory processes that may occur after brain damage as well as the diverse lesion ages that have been studied further complicate efforts to achieve a consistent interpretation of existing work. Explaining these differences across lesion studies and saccade paradigms is therefore methodologically difficult. Additionally, as lesion studies up to date have mainly investigated saccades on the horizontal plane, data about saccadic control on the vertical plane is insufficient and limited to single case reports (Pflugshaupt et al., 2008; Kaiboriboon et al., 2012).

Overcoming some of the limitations of lesion studies outlined above, non-invasive brain stimulation techniques can temporarily modulate neural excitability levels within localized brain regions and have proven their ability of testing the causal role of a given brain area for a certain function in a controlled experimental setting. Somewhat surprisingly, TMS studies targeting FEF have so far not succeeded in producing more consistent results than lesion studies.

Using online TMS paradigms to investigate reflexive saccades with a gap task, single TMS pulses significantly increased reflexive saccade latencies toward the contralateral hemifield when TMS was applied over FEF in the gap period of the task (Nagel et al., 2008). Yet, when TMS was applied simultaneously with target onset, saccade latencies decreased, whereas when TMS was applied during saccade preparation, saccade latencies increased instead (Nyffeler et al., 2004). In addition to online TMS studies, also repetitive TMS designs have been used to study the functional role of FEF in saccade behavior. Using a classical 1 Hz rTMS protocol over right FEF led to significantly increased latencies for visually triggered saccades in both hemifields (Nyffeler et al., 2006c); a finding that was replicated in two follow-up experiments using a modified protocol of continuous theta burst stimulation (cTBS) (Nyffeler et al., 2006a,b).

In the context of voluntary saccades, single TMS pulses were often applied during the preparatory period, revealing significant increases in saccade latency (Thickbroom et al., 1996; Ro et al., 1997, 1999, 2002). To the best of our knowledge, there are no studies examining voluntary saccades triggered by endogenous cues with an offline TMS approach. Although there is a conception that FEF is involved to a greater extent in voluntary saccades, whereas the parietal eye fields contribute to reflexive saccades (Muri and Nyffeler, 2008), the evidence above suggests that FEF is also instrumental in the generation of reflexive saccades. However, the exact role of FEF in voluntary vs. reflexive saccades has not been directly addressed in TMS research.

In sum, it currently seems impossible to provide a coherent integration of findings from TMS studies, mirroring the situation described from lesion studies. Their interpretation is particularly complicated because a variety of factors prevent a direct comparison of studies, such as differences in task parameters that emphasize distinct aspects of saccade generation, and differences in TMS methodology (localization approach, pulse timing, control conditions). In order to overcome these problems, we set out to investigate critical aspects of FEF function in a full within-subject design. Using functional magnetic resonance imaging-guided TMS, we created “virtual lesions” in either left or right FEF (compared to sham TMS) to reveal hemispheric asymmetries in both voluntary and reflexive saccade generation toward horizontal as well as vertical targets. We aimed to directly compare these processes and to uncover dissociations between them. Keeping parameters as similar as possible across tasks, and alternating between tasks before and after the application of TMS, ensured that potential dissociations could be clearly attributed to differential involvement of FEF in these distinct aspects of saccade generation. However, as will be shown below, TMS did not cause any effect on saccade latencies across all experimental conditions.

## Materials and Methods

### Participants

Eighteen healthy participants were recruited from the Maastricht University community. All were right handed, had normal or corrected-to-normal vision, and had no history of any neurological disease or psychiatric disorder. Handedness was determined using a modified version of the 10-item Edinburgh Handedness Questionnaire (Oldfield, 1971; Cohen, 2008), leading to exclusion of one participant due to a very low laterality index score. One additional participant was later excluded from data analysis due to inadequate task execution (see *Statistical analysis*), resulting in a final sample of sixteen participants (mean age = 23.2, 4 male). Before study participation, written informed consent was obtained and participants were screened for TMS experimentation safety. The study was approved by the ethics committee of the Faculty of Psychology and Neuroscience at Maastricht University.

### Overall Study Design

We used a full within-subject design to assess the effects of TMS on two separate voluntary and reflexive saccade tasks. While one task emphasized the voluntary execution of saccades (A) based on central symbolic cues while the other task relied more on reflexive saccades (B) based on the appearance of peripheral targets guiding saccades, potentially providing means for the demonstration of a dissociation between these two different processes after FEF virtual lesions. Participants were tested in three separate sessions (at least 2 days apart), always following the same procedure but with different TMS conditions (right FEF, left FEF, sham). In all sessions, we assessed task performance through saccade latency (time interval between cue onset and saccade for voluntary saccades, and time interval between target onset and saccade for reflexive saccades) before and after the application of TMS. TMS was based on existing individual fMRI data and guided by a neuronavigation system. Saccade latency prior to TMS (15 min; one block of each task) served as baseline allowing the estimation of TMS effects within each session. Saccade latency after TMS was measured over the course of 30 min (four blocks in total), alternating between the two tasks. The order of sessions and tasks was counterbalanced across participants (AB-TMS-ABAB or BA-TMS-BABA).

### Stimuli and Tasks

**FIGURE 1:**
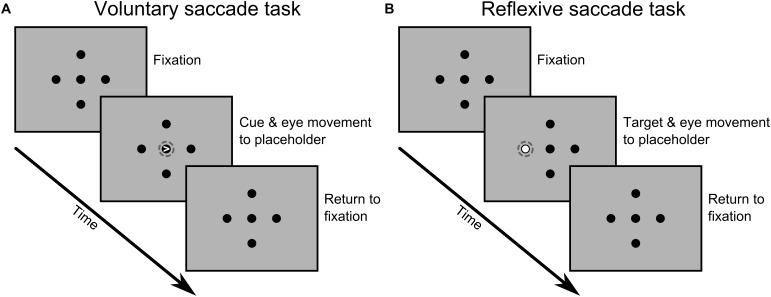
Sequence of events for one possible trial during the voluntary **(A)** and reflexive **(B)** saccade tasks. Visual stimuli and distances are not to scale and the dashed circles were added here to highlight the critical event that led to a saccade to one of the four peripheral locations. A detailed description of the timing of events and task parameters is given in the main text.

The two tasks were designed to differ only with respect to the type of saccade being performed (voluntary vs. reflexive) while keeping other parameters as similar as possible. In both cases, a central fixation dot and four peripheral placeholders were continuously presented on the screen, all subtending 1 degree of visual angle (Figure 1). The placeholders were shown at 8 degrees eccentricity on the horizontal and vertical meridian.

In the voluntary saccade task, we used central symbolic cues to prompt saccades toward peripheral placeholders. Saccades were purely driven by the information provided by the cues thus strongly emphasizing voluntary control mechanisms. Specifically, on each trial a small white arrowhead appeared within the fixation dot for 100 ms, pointing in the direction of one of the four black placeholders. Participants were requested to look at the indicated placeholder as fast as possible and to ensure stable fixation on the target. Then, participants returned to central fixation at their own pace before the next trial started (random inter-trial-interval of 1.5, 2.0, or 2.5 s).

In the reflexive saccade task, we used non-symbolic peripheral cues instead, thus relying more on reflexive control mechanisms. Specifically, one of the four black placeholders was highlighted with a white circle displayed inside the placeholder for 1 s. Again, participants were requested to look at the indicated placeholder as fast as possible and to ensure stable fixation on the target. Once the peripheral placeholder returned to its original color, participants returned to central fixation awaiting the next trial (same inter-trial-interval as above).

At the beginning of each session, participants received written instructions about both tasks and performed a few practice trials to get used to the task demands and timing of events. Then, participants completed three blocks of each task, one before and two after the application of TMS. Each block lasted approximately 6.5 min and consisted of 128 trials, resulting in 32 trials per target positions for each time point (pre, post1, and post2). The order of trials was randomized, but we ensured that all target positions occurred four times in a set of sixteen trials.

Stimuli were presented on an Iiyama ProLite monitor at 57 cm viewing distance. The video mode was 1280 × 1024 at 60 Hz, and background luminance was 100 cd/m^2^. The Presentation software package (NeuroBehavioural Systems, Albany, CA, United States) was used to control stimulus presentation and to send triggers to the eye tracking equipment.

### Localization of TMS Targets

Individual neuroimaging data was available for all participants, obtained during earlier fMRI studies (unpublished) using a Siemens 3T MAGNETOM Prisma Fit scanner at the Scannexus facility in Maastricht, Netherlands^1^. The procedures were essentially identical to those previously described by our lab (Duecker et al., 2013), but scanning parameters and stimulus timing slightly differed across participants. In all cases, functional data were obtained with good spatial and temporal resolution (voxel size: ≤3 mm isotropic, TR: ≤1100 ms), and projected on high-resolution anatomical data (voxel size: 1 mm isotropic). Participants completed an FEF localizer task, lasting about 5 min, consisting of alternating blocks of central fixation and saccadic eye movements toward eight predefined locations along the horizontal and vertical meridian. The localizer task was thus very similar to the saccade tasks used in the present study, ensuring precise localization of task-relevant FEF clusters in both hemispheres. The fMRI data was analyzed using the BrainVoyager software package (Brain Innovation, The Netherlands), following standard procedures with default settings. A simple general linear model contrasting blocks of central fixation and saccades revealed robust BOLD signal changes near the junction of the precentral sulcus and superior frontal sulcus in all participants. The most significant cluster in that area was defined as FEF in each hemisphere and individual coordinates in volume space were later used for TMS coil positioning. Mean Talairach coordinates for left FEF and right FEF were (*x* = −28.2 ± 6.4, *y* = −6.8 ± 5.3, *z* = 50.7 ± 6.4) and (*x* = 34.1 ± 6.4, *y* = −5.9 ± 4.5, *z* = 50.8 ± 5.9), respectively.

### Transcranial Magnetic Stimulation

We followed the same TMS procedures in all three sessions, using a MagVenture MagPro X100 stimulator, equipped with an MC-B70 figure-of-eight coil for active stimulation, and an MC-P-B70 placebo coil for sham stimulation (MagVenture, Farum, Denmark).

We first determined the resting motor threshold over right motor cortex, defined as the minimum intensity that elicited an observable muscle twitch in three out of six trials. The motor threshold of the first session was used as a reference for the stimulation intensity in all sessions. The thresholds of later sessions were only obtained to monitor potential excitability changes across sessions. As expected, no systematic differences were observed between sessions [*F*_(2, 14)_ = 1.304, *p* = 0.286] with the mean motor threshold being practically the same across the first, second, and third session (33.5 ± 4.7%, 32.6 ± 4.5%, and 32.9 ± 4.4% of maximum stimulator output, respectively).

In order to decrease cortical excitability of FEF (virtual lesion), we applied cTBS for 40 s (600 pulses; 50 Hz triplets in a 5 Hz rhythm; at 80% resting motor threshold. TMS coil positioning was based on individual fMRI data, as outlined above, and assisted by the Localite TMS Navigator system (Localite GmbH, Sankt Augustin, Germany) ensuring accurate targeting of FEFs in all participants. The TMS coil was placed with an orientation of 45 degrees to the mid-sagittal plane (handle pointing posteriorly), so that the induced current of the biphasic TMS pulses was strongest in the lateral to medial direction, approximately following the direction of the precentral sulcus. Based on the exact coordinates of FEF and tracking data of the TMS coil, we could also determine the individual scalp-to-cortex distance of our TMS targets. No systematic difference was found between left and right FEF [*t*_(15)_ = 0.019, *p* = 0.985], with an average distance of 25.8 mm in both hemispheres.

For sham stimulation, the placebo TMS coil was aimed at either left or right FEF (pseudo-randomized across participants), and due to the attenuation of the magnetic field, none or very minor TMS effects should be expected in this condition, while matching many of the unspecific aspects of active TMS (Duecker and Sack, 2015).

### Eye Tracking

An EyeLink 1000 Plus system with a desktop mount (SR Research Ltd., Canada) was used to record eye movement data from the dominant eye (left eye in seven and right eye in nine participants). The sampling rate was 1000 Hz and the parameters from a nine-point calibration procedure were accepted when gaze position accuracy was within 1 degree of visual angle for all targets. Saccades and blinks were automatically detected by the eye tracker software and triggers received from the stimulus PC indicated the onset of saccade cues/targets in the eye tracker logfiles. This allowed extraction of relevant saccade parameters for each trial, in particular saccade amplitude and latency.

### Statistical Analysis

Saccades starting with a deviation greater than one degree of visual angle from the central fixation point and saccades failing to end within two degrees of visual angle from the correct target location were excluded from the analysis. Trials with saccade latencies below 80 ms were considered to be anticipatory and were excluded as well. Then, high and low latency outlier values were detected for each experimental condition using the interquartile range (IQR) method and excluded according to the 1.5 × IQR criterion. Based on the total number of excluded trials, one participant was removed from further analysis due to inadequate task execution, that is, nearly half of the trials (47%) failed to meet the criteria described above. This was in clear contrast to the remaining sample where only 19.4% of trials were excluded (7170 out of 36864 trials).

Mean saccade latencies were calculated for each block (pre, post-1, post-2) and separate repeated-measures ANOVAs were performed for reflexive and voluntary saccade tasks. For all analyses, we used a combination of the factors TMS (right FEF, left FEF, sham), Time (pre, post-1, post-2), and Saccade Direction (left, right, up, down) as within-subject factors. *Post hoc* analyses were done using *t*-tests with corrections for multiple comparisons as indicated in the results section. These analyses were computed using SPSS v23 (IBM Corp., Armonk, NY, United States). Additionally, complementary Bayesian repeated-measures ANOVAs (Rouder et al., 2012; Wagenmakers et al., 2018a,b) focusing on the change from baseline for the time period immediately after application of TMS (pre minus post-1) were conducted using the JASP software package (version 0.9) with default prior scales (JASP Team, 2018). Unlike conventional significance testing, this allows determining the evidence in favor of a null result which can be particularly relevant when TMS seemingly had no effect (Biel and Friedrich, 2018; de Graaf and Sack, 2018).

## Results

### Baseline Saccade Latency

We first explored baseline performance on the voluntary and reflexive saccade task, that is, prior to the application of TMS. To begin with, we assessed whether our inclusion of practice trials was sufficient to ensure stable saccade latencies at the beginning of each session, and in how far counterbalancing successfully prevented baseline differences between TMS conditions. To this end, we split the baseline block in half, averaged the saccade latencies across saccade directions, and conducted a repeated-measures ANOVA with TMS (right FEF, left FEF, sham) and Time (1st half, 2nd half) as within-subject factors.

For the voluntary saccade task, this analysis showed no significant main effects [TMS: *F*_(2, 30)_ = 0.37, *p* = 0.69; Time: *F*_(1, 15)_ = 0.44, *p* = 0.52] or interaction [*F*_(2, 30)_ = 2.25, *p* = 0.12], thus indicating that saccade latency was stable throughout the baseline block and, as expected, the same for all TMS conditions. In contrast, the same analysis for the reflexive saccade task revealed a significant interaction between TMS and Time [*F*_(2, 30)_ = 3.56, *p* = 0.04], but no main effects [TMS: *F*_(2, 30)_ = 0.19, *p* = 0.83; Time: *F*_(1, 15)_ = 0.23, *p* = 0.63]. The interaction was clearly driven by higher saccade latencies in the sham TMS condition during the first half of the baseline block whereas all other conditions were indistinguishable. *Post hoc* paired *t*-tests showed that the difference between the first and second half was significantly greater in the sham sessions compared to the right FEF [*t*_(15)_ = 2.20, *p* = 0.044] and left FEF sessions [*t*_(15)_ = 2.45, *p* = 0.027]. Further inspection of the data on the individual level showed that this effect could be traced back to a few participants who received sham TMS during their first session, and they only converged to the performance level of the entire group during the second half of the baseline block. For that reason, we decided to exclude the first half of the baseline block from all subsequent analyses of the reflexive saccade task.

**FIGURE 2:**
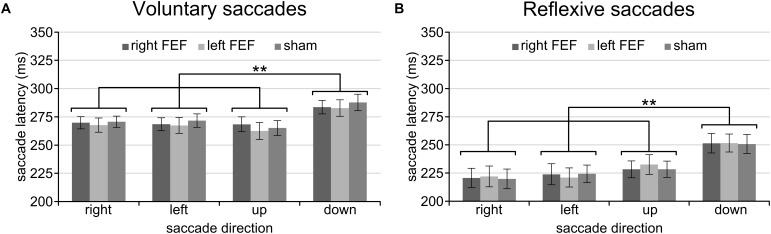
Mean saccade latencies of the voluntary **(A)** and reflexive **(B)** saccade tasks during the baseline block. In line with previous studies, downward saccades had longer saccade latencies than all other saccade directions. The asterisks indicate a significant difference at an alpha level of 0.01 (^∗∗^), and error bars represent standard errors of the mean.

Having established a stable baseline for each task, we were then interested in saccade latency differences between saccade directions. A repeated-measures ANOVA with saccade direction (left, right, up, down) and TMS (right FEF, left FEF, sham) as within-subject factors revealed qualitatively identical results for the voluntary and reflexive saccade task (Figure 2). In both cases, we found a significant main effect of Saccade Direction [voluntary: *F*_(3, 45)_ = 18.38, *p* < 0.001; reflexive: *F*_(3, 45)_ = 20.24, *p* < 0.001], replicating a well-documented effect in the eye movement literature (see Discussion). Specifically, *post hoc* paired *t*-tests (Bonferroni-corrected per task) showed that saccade latencies for downward saccades were significantly higher compared to all other directions (all *p*-values < 0.01), whereas none of the other comparisons between saccade directions reached significance. There was no main effect of TMS [voluntary: *F*_(2, 30)_ = 0.37, *p* = 0.69; reflexive: *F*_(2, 30)_ = 0.20, *p* = 0.98], and no interaction between TMS and Saccade Direction [voluntary: *F*_(6, 90)_ = 1.09, *p* = 0.37; reflexive: *F*_(6, 90)_ = 0.29, *p* = 0.94], establishing that potential TMS effects in subsequent analyses could not be driven by baseline differences.

Lastly, we also evaluated the overall saccade latencies on the voluntary and reflexive saccade task by averaging across all factors. As expected, voluntary saccades (*M* = 272.2 ms, *SD* = 22.1) had increased latencies compared to reflexive saccades (*M* = 231.3 ms, *SD* = 28.1) on the group level [*t*_(15)_ = 9.45, *p* < 0.001], and this pattern was observed in all participants. This behavioral difference strongly suggests that the two saccade tasks indeed emphasized different aspects of saccade generation, supporting their intended conceptual interpretation (voluntary vs. reflexive processes).

### TMS Effects on Saccade Latencies

Mean saccade latencies on the voluntary (Table 1) and reflexive saccade tasks (Table 2) were analyzed using repeated-measures ANOVAs with TMS (right FEF, left FEF, sham), Saccade Direction (left, right, up, down), and Time (pre, post-1, post-2) as within-subject factors.

**Table 1:** Voluntary saccade latency (in milliseconds) and standard error of mean (in parenthesis) for all conditions.

	Right FEF			Left FEF			Sham		
	Pre	Post-1	Post-2	Pre	Post-1	Post-2	Pre	Post-1	Post-2
Right	269.8 (5.36)	266.4 (5.56)	264.5 (5.76)	267.7 (6.29)	260.5 (5.89)	263.6 (6.83)	270.7 (5.01)	265.5 (5.70)	266.5 (5.44)
Left	268.5 (5.70)	265.3 (6.77)	263.8 (6.86)	267.4 (6.97)	262.1 (7.16)	266.1 (6.55)	271.7 (5.97)	265.7 (7.04)	269.0 (6.14)
Up	268.4 (6.55)	259.0 (6.99)	261.8 (7.07)	262.5 (7.56)	258.3 (6.67)	261.1 (7.74)	265.2 (6.55)	259.4 (7.17)	264.9 (6.60)
Down	283.6 (6.00)	279.2 (6.89)	281.3 (7.33)	282.8 (7.30)	273.7 (7.06)	281.4 (8.09)	287.7 (7.33)	284.2 (8.04)	286.6 (7.04)

**Table 2:** Reflexive saccade latency (in milliseconds) and standard error of mean (in parenthesis) for all conditions.

	Right FEF			Left FEF			Sham		
	Pre	Post-1	Post-2	Pre	Post-1	Post-2	Pre	Post-1	Post-2
Right	220.6 (8.57)	218.4 (7.28)	219.6 (6.95)	222.1 (9.19)	213.5 (9.37)	221.6 (9.73)	219.9 (8.76)	214.3 (7.56)	219.8 (7.40)
Left	224.0 (9.27)	221.0 (8.46)	221.5 (8.08)	221.1 (8.50)	217.3 (10.43)	219.6 (9.71)	224.3 (7.83)	215.9 (6.89)	223.8 (9.65)
Up	228.3 (7.44)	222.8 (8.46)	231.2 (6.57)	232.6 (8.69)	225.6 (7.88)	229.3 (8.09)	228.5 (7.17)	221.4 (6.07)	228.3 (7.22)
Down	251.5 (8.68)	244.7 (8.82)	250.5 (8.32)	251.6 (7.96)	244.3 (7.27)	254.6 (8.91)	250.8 (8.51)	247.3 (8.80)	249.5 (8.91)

**FIGURE 3:**
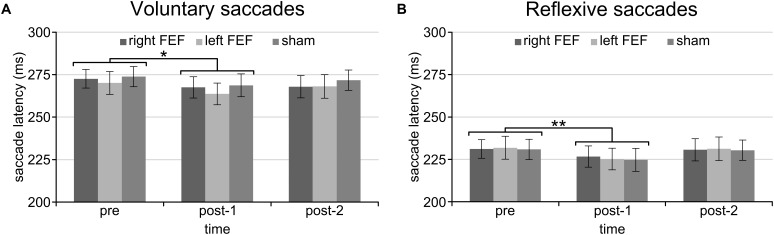
Mean saccade latencies of the voluntary **(A)** and reflexive **(B)** saccade tasks for the entire session. Saccade latencies were decreased immediately after the application of TMS compared to the baseline block. The asterisks indicate a significant difference at an alpha level of 0.05 (^∗^) or 0.01 (^∗∗^), and error bars represent standard errors of the mean.

For both saccade tasks, this revealed significant main effects of Saccade Direction [voluntary: *F*_(4, 45)_ = 24.01, *p* < 0.001; reflexive: *F*_(3, 45)_ = 25.49, *p* < 0.001] and Time [voluntary: *F*_(2, 30)_ = 8.18, *p* = 0.001; reflexive: *F*_(2, 30)_ = 5.81, *p* = 0.007], which were further explored with *post hoc* paired *t*-tests (Bonferroni-corrected per task). In keeping with the baseline data, downward saccades had increased latencies compared to all other directions on both tasks (all *p*-values < 0.001). Moreover, saccade latencies at “post-1” were significantly reduced compared to “pre” [voluntary: *t*_(15)_ = 4.72, *p* < 0.001; reflexive: *t*_(15)_ = 3.26, *p* = 0.015]. This decrease in saccade latency was very small (mean difference < 6 ms), and is probably related to within-session practice effects or higher arousal levels immediately after the application of TMS (Figure 3).

**FIGURE 4:**
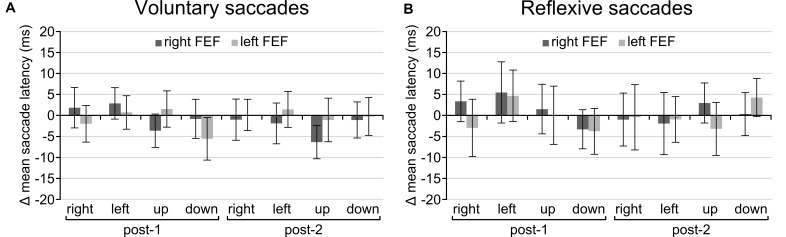
Difference in scores after baseline correction and subtraction from the sham TMS condition for the voluntary **(A)** and reflexive **(B)** saccade tasks. None of the conditions were significantly different from zero, indicating that TMS had no effect on saccade latencies. Error bars represent standard errors of the mean.

Regarding the main goal of the experiment, the main effect of TMS and all interactions failed to reach significance. There was no main effect of TMS [voluntary: *F*_(2, 30)_ = 0.814, *p* = 0.453; reflexive: *F*_(2, 30)_ = 0.033, *p* = 0.968], no interaction between TMS and Saccade Direction [voluntary: *F*_(6, 90)_ = 1.21, *p* = 0.356; reflexive: *F*_(6, 90)_ = 0.506, *p* = 0.803] or Time and TMS [voluntary: *F*_(4, 60)_ = 0.493, *p* = 0.741; reflexive: *F*_(4, 60)_ = 0.126, *p* = 0.972], and the critical three-way interaction between TMS, Time, and Saccade Direction was also not significant for both tasks [voluntary: *F*_(12, 180)_ = 1.057, *p* = 0.399; reflexive: *F*_(12, 180)_ = 0.670, *p* = 0.778]. In other words, there is no statistical support that TMS over FEF had any effect on saccade latencies on the voluntary and reflexive saccade task. Even on a descriptive level, saccade latencies in the active TMS conditions were indistinguishable from sham TMS for all saccade directions (Figure 4).

### Complementary Bayesian Repeated-Measures ANOVA

Given the absence of significant TMS effects described above, we wondered how strong the evidence in favor of a null result was. This cannot be determined by conventional significance testing, and we therefore conducted Bayesian repeated-measures ANOVAs using default prior scales for the voluntary and reflexive saccade tasks. In these complementary analyses, we focused on the change from baseline for the time period immediately after application of TMS (pre minus post-1) when TMS effects were most likely to occur. We thus only included TMS (right FEF, left FEF, sham) and Saccade Direction (left, right, up, down) as within-subject factors, thereby reducing the number of models and avoiding repeated testing of the main effects of Time and Saccade Direction already established in previous analyses.

For the voluntary and reflexive saccades tasks, the null model clearly outperformed all other models. The Supplementary Material includes a detailed overview of the model comparisons and analyses of effects. Most importantly, inclusion Bayes factors based on matched models for the main effect of TMS and the interaction between TMS and Saccade Direction ranged between 0.039 and 0.093, providing strong evidence in favor of the null model (Wetzels et al., 2011). Our data thus supports a genuine absence of TMS effects on saccade latencies.

## Discussion

The aim of this study was to reveal hemispheric asymmetries between left and right FEF in voluntary and reflexive saccade generation toward horizontal and vertical targets using an fMRI-guided TMS-induced “virtual lesion” approach. Various research lines have convincingly demonstrated a causal role of FEF in saccade generation. Yet, the present study fails to find any effect of brain stimulation on saccade latencies.

### Methodological Considerations

The complete absence of TMS effects is certainly surprising given that FEF is a core node of the oculomotor system. Since the meaningfulness of null results in TMS research can be difficult to establish (de Graaf and Sack, 2011), we will first discuss methodological considerations before proceeding to interpretation of our results. To begin with, we localized FEF using individual fMRI activation foci with an established paradigm contrasting blocks of central fixation and eye movements (Frost and Goebel, 2012). Based on these individual FEF coordinates, we positioned the TMS coil on the optimal scalp position guided by a state-of-the-art neuronavigation system. It thus seems highly unlikely that we failed to properly target FEF.

We then applied an established TMS protocol that has consistently been shown to affect cortical excitability in human primary motor cortex (Huang et al., 2005), and has previously been successfully used over FEF to disrupt covert attention shifts (Duecker et al., 2013; Jaun-Frutiger et al., 2013; Cameron et al., 2015; Cazzoli et al., 2015; Marshall et al., 2015). While this exact TMS protocol has not been used over FEF in the context of saccade generation, earlier studies used conceptually similar inhibitory TMS protocols with significant effects on saccade latencies. For example, Nyffeler et al. (2006b,c) repeatedly found changes of saccade latencies using either 1 Hz rTMS or a modified cTBS protocol with 30 Hz triplets (instead of 50 Hz triplets used here). In motor cortex, the latter protocol has been reported to produce stronger suppression and to display less variability between subjects (Goldsworthy et al., 2012). While such minor differences between studies should certainly not be overlooked, these are unlikely to be the primary reason for the complete absence of TMS effects reported here. While classical cTBS parameters might not lead to optimal effects, it remains a widely used and established protocol that can be expected to have an effect on cortical excitability.

Taken together, the methodological rigor with which we designed and conducted the present TMS study makes it unlikely that we entirely failed to affect FEF on the neurophysiological level. Yet, it has to be noted that we cannot provide data to demonstrate and thus validate the neural efficacy of our TMS protocol (de Graaf and Sack, 2011). Assuming that our TMS methodology indeed caused excitability changes in FEF, it could still be argued that the voluntary and reflexive saccade tasks were insensitive to modulation by our experimental factors. In our view, there are two strong arguments against that notion, both based on the actual saccade latency data obtained in our study. First, there were pronounced saccade latency differences between the voluntary and reflexive saccade tasks compatible with previous studies (Fischer, 1987; Walker et al., 2000). Saccade latencies were clearly longer for voluntary saccades, consistent with the task requirements to either voluntarily initiate a saccade based on a centrally presented arrow vs. a reflexive saccade triggered by a peripheral target. Second, we also replicated a well-known effect of target position on saccade latency, that is, saccade latencies were clearly longer for downward saccades, suggested to be explained through an upper hemifield bias (Dafoe et al., 2007; Abegg et al., 2015). Taken together, these behavioral effects show that saccade latency on both tasks was susceptible to our experimental manipulations.

### Absence of Behavioral Effects

The considerations above point toward a meaningful null result, that is, a “virtual lesion” over FEF does not necessarily change saccade latencies. As mentioned above, this is in clear contrast to previous studies that reported TMS-induced changes of saccade latencies, albeit with quite heterogeneous results (see Introduction). In the following, we will discuss possible explanations for this absence of behavioral effects in relation to these studies and the functional role of FEF, keeping in mind the general interpretative challenges of null results in TMS research.

#### Effect of Placeholders on Oculomotor Network

A key objective of our study was to investigate the involvement of FEF in voluntary and reflexive saccades. We therefore designed two tasks that only differed with respect to the type of saccade being performed while keeping other parameters as similar as possible. Importantly, this required the presentation of placeholders at four target locations (right, left, up, down). In our case, these placeholders were solidly filled circles at a fixed eccentricity, thus providing information on the required saccade end point at all times. Previous TMS studies on reflexive saccades typically did not use placeholders at all (Nyffeler et al., 2006a,b,c), whereas previous TMS studies on voluntary saccades used “empty” squares that only provided minimal visual input around the saccade target (Ro et al., 1997, 1999, 2002).

We speculate that the constant presence of rather salient placeholders in our study might have protected (visually responsive) areas within the oculomotor network from the effects of FEF disruption. According to the stochastic accumulator model of perceptual decision making and action (Gandhi and Katnani, 2011; Schall, 2015), changes of saccade latency can result from changes in baseline activity, accumulation rate, accumulation onset, and saccade threshold. Research in non-human primates has revealed that FEF inactivation disrupts all of the above parameters, but changes of saccade latency only correlated with the delay of accumulation at the level of the superior colliculi (Peel et al., 2017). The superior colliculi are a major relay station between sensory and motor areas, receiving direct input from the retina and early visual cortex (May, 2006), and they are strongly interconnected with cortical areas of the oculomotor network including FEF. The constant display of placeholders might thus have caused activation of the superior colliculi before cue/target onset. This pre-activation could have overruled TMS-induced changes in baseline activity and counteracted potential accumulation delays. In sum, seemingly minor difference in the implementation of placeholders might have resulted in a strong effect on the state of the oculomotor network and its susceptibility to TMS-induced disruption.

#### Overall Low Cognitive Demand

An alternative reason for the absence of TMS effects in our study might be the overall low cognitive demand required to execute the tasks. There are various classes of saccade paradigms (gap, overlap, anti-saccades, etc.) and we intended to circumvent any complexities that might affect the comparison between voluntary and reflexive saccade in the horizontal and vertical direction. Yet, this led to a reduced task difficulty compared to previous studies. In our reflexive saccade task, several factors contributed to the relatively low task demands. First, the presence of placeholders might have facilitated target selection. Second, the constant eccentricity of targets might have simplified saccade planning and preparation. Third, targets were presented for quite a long time, potentially guiding the saccade toward the end point. All of these points are in contrast to work by Nyffeler et al. (2006a,b,c), who repeatedly found TMS effects on reflexive saccade latency, where the eccentricity was unpredictable and targets were displayed very briefly (80 ms), thus increasing cognitive demand. In our voluntary saccade task, essentially the same arguments apply. The presence of placeholders at a constant eccentricity potentially allowed participants to engage in preparatory activities prior to cue presentation, since only the saccade direction was contingent on the cue. Moreover, some studies relied on auditory cues instead, which further increases task demands (Priori et al., 1993; Thickbroom et al., 1996). In sum, the low cognitive demand of our tasks might have resulted in a reduced involvement of higher cortical control regions such as FEF, potentially leaving sufficient resources intact after FEF disruption.

#### No Net Effect of TMS

FEF has been implicated in many aspects of saccade generation and fixation control and strongly interacts with other nodes of the oculomotor network (Matsumoto et al., 2018). Consequently, the use of an inhibitory TMS protocol over FEF most likely influences various processes that are relevant for saccade tasks. From a theoretical perspective, it is conceivable that our TMS protocol indeed disrupted FEF, but had no observable effect due to the functional diversity of FEF. Specifically, we intended to disrupt processes related to saccade generation, hypothesizing that this would lead to increased saccade latencies, but a simultaneous disruption of fixation control might have facilitated fixation disengagement resulting in decreased saccade latencies, thus leading to the absence of a net effect. Similarly, a TMS-induced decrease of cortical excitability over FEF can be accompanied by disinhibition of the superior colliculi (Pierrot-Deseilligny et al., 1987), again allowing for opposite changes of saccade latency. Obviously, such an unfortunate combination of additive or cancelation effects is rather unlikely to produce a null result across all conditions. Yet, we consider this an important consideration as complex TMS effects might remain hidden on the behavioral level.

#### Offline vs. Online TMS

Given our research question, using an offline TMS approach seemed most promising, because it ensured that the TMS-induced changes in FEF were identical for both tasks. Yet, the majority of TMS studies investigating the role of FEF in saccade generation opted for the use of online TMS instead. That is, they applied TMS pulses at specific time points during the saccade generation process. We reasoned that deciding on the exact timing of these TMS pulses is practically impossible when the goal is to compare two tasks with different saccade latencies, but the use of offline TMS is not without problems. There are several reasons why an offline TMS protocol might fail to cause behavioral effects; a few have already been hinted at above. We here stress a few additional issues. First, it could be argued that effects of offline TMS are weaker compared to the immediate disruption of TMS pulses delivered at the optimal period of relevant processing. It might very well be that the impact of our TMS protocol on the neurophysiological level was simply too weak to have an effect on the behavioral level. Second, offline TMS approaches have a higher probability of allowing compensatory brain mechanisms to occur (Sack et al., 2005). The brain might adapt to the TMS-induced changes in cortical excitability by recruiting more or other neuronal resources. To illustrate, in the light of the stochastic accumulator model outline above, TMS might successfully reduce the baseline activity or accumulation rate, but a simple change in saccade threshold might counteract these effects. Third, offline TMS studies typically require multiple sessions on different days. In order to reduce variability due to session effects, we used a pre/post design to have within-session baselines. Yet, studies in the human motor cortex have convincingly shown that the effects of offline TMS can depend on the current brain state, a process referred to as metaplasticity (Gentner et al., 2008; Iezzi et al., 2008; Goldsworthy et al., 2014). Executing the saccade tasks prior to the application of TMS could in principle have affected the magnitude and/or direction of our TMS effect.

Looking forward, the present results seem to suggest that online TMS approaches are more promising to further investigate the role of FEF in saccade generation. They certainly pose greater methodological challenges, and studies thus far have employed diverging tasks and TMS parameters. Yet, they have the potential of disentangling the various contributions of FEF in the oculomotor network, in particular when using fully chronometric designs.

## Conclusion

Despite using a state-of-the-art TMS approach, we convincingly failed to observe any TMS effect on saccade latency, as indicated by conventional and Bayesian analyses. We have identified various factors that might have contributed to the absence of effects, but these issues remain speculative at this point. Nonetheless, research on the role of FEF in saccade generation should carefully consider seemingly subtle task parameters such as the use of placeholders and cognitive demand, as well as the difficulties that arise when targeting a network with complex interactions and multiple functional roles. To conclude, we hope that future studies will create a context where the theoretical meaningfulness of our null result is established, and not just considered a failed experiment.

## Ethics Statement

This study was carried out in accordance with the recommendations of tCs and TMS safety protocol and Code of Ethics for research in the Social and Behavioral Sciences involving human subjects, Ethics Review Committee Psychology and Neuroscience (ERCPN) with written informed consent from all subjects. All subjects gave written informed consent in accordance with the Declaration of Helsinki. The protocol was approved by the Ethical Review Committee of the Faculty of Psychology and Neuroscience of Maastricht University, The Netherlands.

## Author Contributions

FD, SG, and AS contributed to methodological design. SG and FD contributed to data acquisition and analysis. All authors contributed to the writing of the manuscript.

## Conflict of Interest Statement

The authors declare that the research was conducted in the absence of any commercial or financial relationships that could be construed as a potential conflict of interest.

## Footnotes

1 www.scannexus.nl
