# Supplementary material for: Assessing the Functional Role of Frontal Eye Fields in Voluntary and Reflexive Saccades Using Continuous Theta Burst Stimulation
Source: Front Neurosci. 2018 Dec 14;12:944. doi: 10.3389/fnins.2018.00944 (PMC6302006; doi:10.3389/fnins.2018.00944)
Supplement: Supplementary file 1 [file Table_1.DOCX]

**Voluntary Saccade Task**

**Bayesian Repeated Measures ANOVA**

| **Model Comparison** | | | | | | | | | | | |
| --- | --- | --- | --- | --- | --- | --- | --- | --- | --- | --- | --- |
| **Models** | | **P(M)** | | **P(M\|data)** | | **BF _M_** | | **BF _10_** | | **error %** | |
| Null model (incl. subject) |  | 0.200 |  | 0.903 |  | 37.376 |  | 1.000 |  |  |  |
| TMS |  | 0.200 |  | 0.064 |  | 0.274 |  | 0.071 |  | 1.101 |  |
| POSITION |  | 0.200 |  | 0.030 |  | 0.125 |  | 0.033 |  | 0.827 |  |
| TMS + POSITION |  | 0.200 |  | 0.002 |  | 0.009 |  | 0.002 |  | 0.996 |  |
| TMS + POSITION + TMS  ✻  POSITION |  | 0.200 |  | 1.978e -4 |  | 7.915e -4 |  | 2.190e -4 |  | 1.100 |  |
|  | | | | | | | | | | | |
| *Note.*  All models include subject. | | | | | | | | | | | |

| **Analysis of Effects** | | | | | | | |
| --- | --- | --- | --- | --- | --- | --- | --- |
| **Effects** | | **P(incl)** | | **P(incl\|data)** | | **BF _Inclusion_** | |
| TMS |  | 0.400 |  | 0.066 |  | 0.071 |  |
| POSITION |  | 0.400 |  | 0.032 |  | 0.033 |  |
| TMS  ✻  POSITION |  | 0.200 |  | 1.978e -4 |  | 0.093 |  |
|  | | | | | | | |
| *Note.*  Compares models that contain the effect to equivalent models stripped of the effect. Higher-order interactions are excluded. Analysis suggested by Sebastiaan Mathôt. | | | | | | | |

**Reflexive Saccade Task**

**Bayesian Repeated Measures ANOVA**

| **Model Comparison** | | | | | | | | | | | |
| --- | --- | --- | --- | --- | --- | --- | --- | --- | --- | --- | --- |
| **Models** | | **P(M)** | | **P(M\|data)** | | **BF _M_** | | **BF _10_** | | **error %** | |
| Null model (incl. subject) |  | 0.200 |  | 0.908 |  | 39.326 |  | 1.000 |  |  |  |
| TMS |  | 0.200 |  | 0.065 |  | 0.277 |  | 0.071 |  | 1.038 |  |
| POSITION |  | 0.200 |  | 0.026 |  | 0.105 |  | 0.028 |  | 0.541 |  |
| TMS + POSITION |  | 0.200 |  | 0.002 |  | 0.007 |  | 0.002 |  | 1.636 |  |
| TMS + POSITION + TMS  ✻  POSITION |  | 0.200 |  | 7.159e -5 |  | 2.864e -4 |  | 7.887e -5 |  | 1.259 |  |
|  | | | | | | | | | | | |
| *Note.*  All models include subject. | | | | | | | | | | | |

| **Analysis of Effects** | | | | | | | |
| --- | --- | --- | --- | --- | --- | --- | --- |
| **Effects** | | **P(incl)** | | **P(incl\|data)** | | **BF _Inclusion_** | |
| TMS |  | 0.400 |  | 0.067 |  | 0.071 |  |
| POSITION |  | 0.400 |  | 0.028 |  | 0.028 |  |
| TMS  ✻  POSITION |  | 0.200 |  | 7.159e -5 |  | 0.039 |  |
|  | | | | | | | |
| *Note.*  Compares models that contain the effect to equivalent models stripped of the effect. Higher-order interactions are excluded. Analysis suggested by Sebastiaan Mathôt. | | | | | | | |
